# Cost of Cerebellar Ataxia in Hong Kong: A Retrospective Cost-of-Illness Analysis

**DOI:** 10.3389/fneur.2020.00711

**Published:** 2020-07-17

**Authors:** Winser John Stanley, Chan Kit Laam Kelly, Chinn Ching Tung, Tang Wai Lok, Tye Man Kit Ringo, Yeung Kai Ho, Raymond Cheung

**Affiliations:** Department of Rehabilitation Sciences, The Hong Kong Polytechnic University, Kowloon, Hong Kong

**Keywords:** cerebellar ataxia, cost-utility, cost of illness, direct cost, indirect cost

## Abstract

**Background:** Cerebellar ataxia affects the coordination and balance of patients. The impact of this disease increases burden in patients, caregivers and society. Costs and the burden of this disease have not been investigated in Hong Kong.

**Objectives:** (1) To estimate the socioeconomic cost of cerebellar ataxia in Hong Kong for the base year 2019, (2) to assess the health-related quality of life (HRQoL) and severity of ataxia, and (3) to establish the correlation between the severity and cost of cerebellar ataxia and to examine the correlation between the severity of cerebellar ataxia and HRQoL.

**Methods:** A retrospective cross-sectional study was conducted amongst 31 patients with cerebellar ataxia. Cost-related data were obtained through self-reported questionnaires. The severity of ataxia was assessed using the Scale for Assessment and Rating of Ataxia, and HRQoL was assessed using the Short Form (36) Health Survey (SF-36). Pearson correlation was used for normally distributed data, whereas Spearman correlation was used otherwise.

**Results:** The mean severity of ataxia was 21 out of 40. The average direct and indirect costs of a patient with ataxia in 6 months were HKD 51,371 and HKD 93,855, respectively. The mean difference between the independent to minimally dependent in activities of daily living (ADL) group and the moderate to maximally dependent in ADL group for direct and indirect costs was HKD 33,829 and HKD 51,444, respectively. Significant expenditure was related to production lost (42%), caregiver salary (17%), and in-patient care (16%). The physical functioning (*r* = −0.58) and general health (*r* = −0.41) of SF-36 were negatively correlated with disease severity (*p* < 0.05). A significant, positive correlation was found between disease severity and direct cost (Spearman's rho = 0.39) and the cost of hiring a caregiver (Spearman's rho = 0.43).

**Conclusion:** The mean cost for 6 months for patients with cerebellar ataxia in Hong Kong is HKD 146,832. Additional support, including employment, access to specialist consultants, informal home care and community participation, are some areas that should be addressed. Future study on a larger population with a prospective design is necessary to confirm the aforementioned claims.

## Introduction

Cerebellar ataxia is not a disease but a collection of symptoms resulting from damage to the cerebellum or its connections (1). This heterogeneous group of disorders can be broadly classified into acquired and hereditary types (1). The cardinal features of ataxia include dyssynergia, dysmetria, tremor, poor balance, gait instability, dysarthria, and cognitive impairment (1, 2). Poor balance and gait instability contribute to the high incidence of injurious falls amongst patients with cerebellar ataxia. Van de Warrenburg et al. reported that 93% of patients with ataxia are fallers, with 85% suffering from injurious falls (3). The international prevalence of cerebellar ataxia ranges from 0.3 to 3.0 per 100,000 (4), depending on the underlying cause. A study conducted in Korea reported a prevalence rate of 8.29 per 100,000 (5).

Most cardinal features of cerebellar ataxia affect patient's ability to resume work; this is likely to increase the economic burden of the individual and society (6). In addition, some patients with cerebellar ataxia require support from family members or paid assistants for their activities of daily living (ADL), increasing the indirect expense due to the condition (6). In Hong Kong, social support for patients with cerebellar ataxia who are eligible citizens includes subsidies for medical expenses, monetary subsidies for disablement and welfare from a self-help group called the Hong Kong Spinocerebellar Ataxia Association (HKSCAA).

To our knowledge, no research that reports the cost of cerebellar ataxia is currently available within the Asian region, including Hong Kong. Therefore, a study that establishes the direct and indirect costs of cerebellar ataxia and the quality of life (QoL) of patients with cerebellar ataxia is necessary. The findings of this study will highlight the demand for care of patients with cerebellar ataxia. Furthermore, the result of this research will inform policymakers about restructuring the needs of patients with cerebellar ataxia, if any. Therefore, the current study aims to (1) estimate the direct and indirect socioeconomic costs of cerebellar ataxia amongst patients with cerebellar ataxia of any cause in Hong Kong, (2) assess the health-related QoL (HRQoL) of patients with cerebellar ataxia in 2019 in Hong Kong, (3a) estimate the correlation between the severity of cerebellar ataxia and cost and (3b) estimate the correlation between the severity of cerebellar ataxia and QoL.

## Materials and Methods

We conducted a retrospective cross-sectional survey-based study. Ethics approval was obtained from the Human Subjects Ethics Committee of the Hong Kong Polytechnic University (HSECS reference number: HSEARS20190524001). This trial was registered with the Chinese Clinical Trials (registration number: ChiCTR1900023440). We used a self-reported questionnaire to obtain information about cost-utility over the past 6 months amongst patients with cerebellar ataxia in Hong Kong for the base year 2019.

Patients were recruited through HKSCAA. Volunteers were included if they had a confirmed diagnosis of cerebellar ataxia of any or unknown cause. They were excluded if they were unwilling to reveal personal information. The purpose of the study and the procedures and potential risks involved in the study were explained in native language. Patients providing written consent were enrolled. A questionnaire requesting information about demographics and socioeconomic cost were distributed to the enrolled patients through HKSCAA. In this study, a fall refers to the rapid uncontrolled change from a sitting or upright position to a reclining or coming to ground position (7), and “frequent fallers” are those who fell five or more times in the past 6 months.

The severity of cerebellar ataxia was assessed using the Scale for the Assessment and Rating of Ataxia (SARA). The scale is scored out of 40. The higher the score, the more severe the ataxic symptoms. The assessment was performed by members of the research team, who were trained by the team supervisor. In this study, our participants were categorized by dependency in ADL based on the overall SARA scores. Patients who scored <10 were categorized into the independent to minimally dependent group, and those who scored >10 were placed under the moderate to maximally dependent group (8). SARA has been reported to have high inter-rater reliability (9), test–retest reliability (9), and construct validity (10) for estimating disease severity in patients with cerebellar ataxia. The Short Form (36) Health Survey (SF-36) was used to measure HRQoL. SF-36 includes eight each scored between 0 and 100, with 0 indicating worst health and 100 indicating the best health status possible. Previous studies have used SF-36 amongst patients with ataxia (11–13). The Chinese version of SF-36 was used in this study. This version exhibits satisfactory convergent and discriminant validities (14, 15). The scale has high test–retest reliability (ICC range 0.66–0.94), and internal consistency (Cronbach alpha range 0.72–0.88) (14).

The patients were asked to recall their medical expenses related to cerebellar ataxia over the past 6 months to estimate socioeconomic cost. The tests were conducted at the patients' residence for those who were unable to reach HKSCAA. The patients answered the 52-item socioeconomic survey; some were assisted by their caregivers or the researchers depending on their ability to write. SF-36 was conducted by the researchers through interviews. The assessment scales were randomly executed. Apart from the assessor, standby assistance was provided by two supporting researchers to ensure safety. The total time required to complete the three tests was 60 min.

We used the cost-of-illness (COI) analysis, also known as the burden of disease to estimate the socio-economic cost. This analysis method considers various effects of health outcomes on a country, specific regions, communities, and even individuals. COI evaluates morbidity in relation to a decrease in health status and QoL and financial aspects, including direct and indirect expenditures resulting from premature death, disability, or injury (16). Such evaluation can identify different components of cost, providing insights into formulating healthcare policies. This information will enable determining funding priorities and allocating healthcare resources by highlighting areas with inefficiencies (17).

We estimated both direct and indirect costs. Direct costs are those incurred by the health system, the society, individual patients, and their family. These cost items include medical care expenditures for diagnosis, treatment, rehabilitation, medical professional services, drugs, and other medical supplies. Indirect costs in COI studies refer to productivity loss due to morbidity and mortality; they are borne by the patient or his/her family (16). In this study, most direct and indirect cost items were obtained in physical units and then converted into monetary units using the human capital method for estimating the value of human capital as the present value of his/her future earnings under the assumption that future earnings are used as a proxy for future productivity (16). The standardized unit costs were obtained from the Hong Kong Hospital Authority Fee and Charges lists (18), and the remaining costs were obtained as monetary units provided by the research patients. Table 1 provides the cost of unsubsidised healthcare-related expenses in Hong Kong for the base year 2019. We used the following criteria to select cost items for this estimation: relevance to ataxia, how quantifiable the items are, how common are the items to be evaluated in economic burden or cost–utility studies and to what extent can they reflect and impact patient's QoL.

**Table 1:** Unsubsidised unit of cost of health and health-related expenditures (in HKD) for the base year 2019 (18).

**Cost items**	**Unit**	**Estimated unit cost (in HKD)**
**Direct healthcare cost**		
1. In-patient care		
1a. General hospital admission due to ataxia	No. of days	$5100
1b. General hospital admission not due to ataxia	No. of days	$5100
1c. Emergency visits due to fall	No. of visits	$1230
1d. Emergency visits not due to fall	No. of visits	$1230
1e. Intensive-care unit (ICU)	No. of days	$24400
2. Out-patient care		
2a. General practitioners for ataxia	No. of visits	$445
2b. General practitioners not for ataxia	No. of visits	$445
2c. Specialist visits for ataxia	No. of visits	$1190
2d. Specialist visits not for ataxia	No. of visits	$1190
2e. Traditional Chinese Medicine for ataxia	No. of visits	$120
2f. Traditional Chinese Medicine not for ataxia	No. of visits	$120
2g. Acupuncture for ataxia for ataxia	No. of visits	$180
2h. Acupuncture not for ataxia not for ataxia	No. of visits	$180
3. Drugs	No. of types of tablet	N/A (*Prices of the specified drugs are according to Lexi-Comp*)
4. Rehabilitation	No. of visits	$1190
5. Walking aids	No. of item	N/A (Prices are according to average costs of five rehabilitation shops in Hong Kong^*^)
6. Miscellaneous	N/A	N/A *(values in monetary units to be collected)*
**In-direct healthcare cost**		
1. Caregiver	No. of month	$4520
2. Transportation	N/A	N/A *(values in monetary units to be collected)*
3. Home modification	No. of items installed	N/A (Prices are referred to average costs of five rehabilitation shops in Hong Kong^*^)
4. Production loss due to disease or early retirements	No. of hour	N/A (*refer to the Hong Kong Census and Statistics Department*)

* *The five leading rehabilitation shops were contacted to estimate the home modification expense. The shops are as follows: (1) Wing Hang Medical Supplies Limited, (2) Just Med Limited, (3) Health Top, (4) Live Smart Rehab, and (5) Healthy Living Medical Supplies Limited*.

### Statistical Analysis

Data analysis was conducted using SPSS version 20. Demographic data were collected and reported in mean and percentage. Descriptive statistics were used to illustrate the socioeconomic costs, disease severity, and QoL of patients with cerebellar ataxia. Inferential statistics were used to report the correlation between disease severity and cost and between disease severity and QoL. If the data were normally distributed, then Pearson correlation was used for quantifying correlation; otherwise, Spearman correlation coefficient was used. In this study, correlation coefficients of 0.3–0.5 were considered low, 0.5–0.7 were moderate, 0.7–0.9 were high and >0.9 were extremely high (19). Moreover, α was set at <0.05 to reject the null hypothesis, i.e., no correlation exists between the factors, including the severity of cerebellar ataxia, cost and QoL.

### Sensitivity Analysis

We conducted two adjusted analysis, first, we excluded participants with acquired cerebellar ataxia such as ataxia secondary to stroke, multiple sclerosis or cerebral palsy as these diseases may have a different clinical course of that of degenerative and hereditary ataxias. Second, we constructed a Tornado plot to geographically represent the degree to which each of the items within the direct and indirect costs influences the total cost of ataxia. The minimum and maximum range of the cost was set at the 25th and 75th percentile and the model was run for each adjusted extreme cost value while keeping the remaining fixed at their mean and median (20, 21).

## Results

This study included 31 patients with cerebellar ataxia aged between 25 and 74 years. Table 2 provides the demographics of the included patients. All of the included patients were the residents of Hong Kong with Chinese ethnical background. A total of 30 had history of falls; amongst them, 87% were frequent fallers (5 or more falls in 6 months). The mean number of falls in 6 months was 26. 90% of the falls were indoor and did not require hospital admission. The mean number of visits to the accident and emergency department (A&E) for falls and falls-related injuries was 1.

**Table 2:** Demographics of the participants (*n* = 31).

Gender	Male		15 (48.4%)
	Female		16 (51.6%)
Age	Mean (SD)		55.1 (14.7)
	Range		25–74
Causes for ataxia	Spinocerebellar ataxia	SCA 3	4 (12.9%)
		SCA 35	1 (3.2%)
		SCA 40	1 (3.2%)
		Unknown type	20 (64.5%)
		Total	26 (83.9%)
	Stroke		1 (3.2%)
	Cerebral palsy		1 (3.2%)
	Unknown cause for ataxia		3 (9.7%)
Diagnosis duration	Mean (SD) in years		15.1 (11.0)
	Range		2–46
Employment status	Full-time		4 (2.9%)
	Part-time		3 (9.7%)
	Self-employed		3 (9.7%)
	Retired		15 (48.4%)
	Unable to work		6 (19.4%)
Education level	No schooling/pre-primary		1 (3.4%)
	Primary school		5 (16.1%)
	Lower secondary		6 (19.4%)
	Upper secondary		7 (22.6%)
	Diploma/certificate		4 (12.9%)
	Degree course		8 (25.8%)
Marital status	Single		13 (41.9%)
	Married		12 (38.7%)
	Divorced/separated		3 (9.7%)
	Widowed		3 (9.7%)
Assistance from a caregiver	Yes		19 (61.3%)
	No		12 (38.7%)
Assistive device for ambulation	Yes		31 (100%)
	No		0 (0%)
Type of assistive device	Electric wheelchair		15 (48.4%)
	Wheelchair		6 (19.4%)
	Frame		1 (3.2%)
	Rollator		2 (6.5%)
	Crutches		0 (0%)
	Quadripod		4 (12.9%)
	Stick		1 (3.2%)
	No walking aids needed		2 (6.5%)
Falls history	Yes		30 (96.8%)
	No		1 (3.2%)
Frequent faller (5 or more falls in 6 months)	Yes		27 (87.1%)
	No		4 (12.9%)
Number of fall	Mean (SD)		26.2 (37.6)
Current housing	Public rental housing		17 (54.8%)
	Private rental housing		2 (6.5%)
	Private permanent housing		10 (32.3%)
	Home for disability		1 (3.2%)
	Home ownership scheme		1 (3.2%)
Living	Alone		2 (6.5%)
	With family		29 (93.5%)
Early retirement	Yes		24 (77.4%)
	No		7 (22.6%)
Disease severity (SARA)	Mean (SD)/40		21.2 (8.2)
	Range		4.5–39.0
Degree of dependence in ADL base on SARA score	Moderate to maximal dependent (SARA <10)		27 (87.1%)
	Independent to minimal dependent (SARA > 10)		4 (12.9%)

Table 3 presents direct and indirect costs due to cerebellar ataxia over the past 6 months. The average cost for each ataxic patient for 6 months was HKD 146,832 (Table 3). Significant expenses accounted for production lost (42%), in-patient care (16%), and caregiver salary (17%). Direct healthcare costs accounted for 35% of the total costs, while indirect costs accounted for 65%.

**Table 3:** Mean cost (SD) [% of total cost] of each patient with cerebellar ataxia over a 6-month duration (in HKD) for the base year 2019.

**Cost items**	**Independent to minimal dependent mean (SD) in HKD {mean visits}**	**Moderate to maximal dependent mean (SD) in HKD {mean visits}**	**Mean difference**	**Total mean (SD) in HKD {mean visits}**
**DIRECT COST**				
1. In-patient care	307.5 (615.0) [0.4%]	26072.2 (61853.7) [16.5%]	25764.7	22747.7 (58248.5) [15.5%]
1a. Hospital admissions due to ataxia	0	17377.8 (59865.7) [10.6%] {3.41}	17377.8	15135.5 (56045.6) [10.0%] {2.97}
1b. Hospital admissions for causes other than ataxia	0	7555.6 (21187.6) [4.6%] {1.48}	7555.6	6580.6 (19891.9) [4.3%] {1.29}
1c. A&E visits due to falls	307.5 (615.0) [0.4%] {0.25}	637.8 (1501.4) [0.4%] {0.52}	330.3	595.2 (1415.7) [0.4%] {1.05}
1d. A&E visits for causes other than falls	0	501.1 (1144.7) [0.3%] {0.41}	501.1	436.5 (1079.2) [0.3%] {0.35}
1e. Intensive care unit (ICU) visit	0	0	0	0
2. Out-patient care	1815.0 (2288.1) [2.6%]	3999.8 (3122.4) [2.5%]	2184.8	3717.9 (3086.6) [2.5%]
2a. GP visits for ataxia	111.3 (222.5) [0.2%] {0.25}	148.3 (445.0) [0.1%] {0.33}	37.0	143.5 (420.4) [0.1%] {0.32}
2b. GP visits for causes other than ataxia	556.3 (1112.5) [0.8%] {1.25}	395.6 (712.6) [0.2%] {0.89}	160.7	416.3 (752.9) [0.3%] {0.94}
2c. SOPD visits for ataxia	595.0 (687.0) [0.8%] {0.50}	1410.4 (935.7) [0.9%] {1.19}	815.4	1305.2 (939.8) [0.9%] {1.10}
2d. SOPD visits for causes other than ataxia	297.5 (595.0) [0.4%] {0.25}	1278.1 (1864.9) [0.8%] {1.07}	980.6	1151.6 (1777.9) [0.8%] {0.97}
2e. TCM visits for ataxia	62.5 (125.0) [0.1%] {0.25}	898.1 (2238.5) [0.5%] {3.59}	835.6	790.3 (2103.7) [0.5%] {3.16}
2f. TCM visits for causes other than ataxia	375.0 (595.1) [0.5%] {1.50}	55.6 (200.2) [0.0%] {0.22}	319.4	96.8 (286.3) [0.1%] {0.39}
2g. Acupuncture for ataxia	62.5 (125.0) [0.1%] {0.25}	444.4 (1368.1) [0.3%] {1.78}	381.9	395.2 (1280.9) [0.3%] {1.58}
2h. Acupuncture for causes other than ataxia	0	37.0 (133.4) [0.0%] {0.15}	37.0	32.3 (124.9) [0.0%] {0.13}
3. Drugs	6670.7 (13341.4) [9.4%]	5574.7 (13200.6) [3.5%]	1096.0	5716.1 (12998.4) [3.9%]
4. Rehabilitation	7140.0 (14280.0) [10.1%] {6.00}	12561.1 (24466.5) [7.9%] {10.56}	5421.1	11861.6 (23293.8) [8.1%] {9.97}
5. Walking aids	3973.5 (7491.4) [5.6%]	8681.5 (7336.5) [5.5%]	4708.0	8074.0 (7405.0) [5.5%]
6. Miscellaneous	2000.0 (4000.0) [2.8%]	652.6 (1079.8) [0.4%]	1347.4	826.5 (1679.7) [0.6%]
Total direct cost	21906.7 (21117.8) [30.9%]	55736.1 (65875.8) [35.3%]	33829.4	51371.0 (62757.5) [35.0%]
**INDIRECT COST**				
1. Caregiver	6780.0 (13560.0) [9.6%]	28124.4 (23160.9) [17.8%]	21344.4	25370.3 (23156.0) [17.3%]
2. Transportation	500.0 (707.1) [0.7%]	412.8 (587.0) [0.3%]	87.2	424.0 (591.2) [0.3%]
3. Home modification	578.0 (719.1) [0.8%]	2372.6 (2462.7) [1.5%]	1794.6	2141.0 (2383.7) [1.5%]
4. Production loss	8733.0 (11408.0) [12.3%]	69584.1 (96057.3) [44.0%]	60850.8	61732.4 (91868.1) [42.0%]
Total indirect cost	49049.5 (54445.5) [69.1%]	100493.9 (109411.3) [63.6%]	51444.4	93855.9 (104778.3) [65%]
Overall expense	70956.2 (52825.2)	158072.9 (146110.9)	87116.8	146832.1 (140222.4)

*A&E, Accident and emergency; OPD, out-patient department; SOPD, Specialized outpatient department; TCM, Traditional Chinese Medicine*.

Amongst the patients in the independent to minimally dependent in ADL group, direct and indirect costs accounted for 31 and 69%, respectively. The patients indicated significant contributions toward expenses for rehabilitation (8%), caregiver salary (10%) and production loss (12%). By contrast, expenses for in-patient care (<1%), transportation (<1%), and home modification (<1%) were insignificant. The patients in the moderate to maximally dependent in ADL group had significant expenses from production loss (44%), in-patient care (17%), and caregiver salary (18%). Transportation (<1%) and home modification (2%) contributed the least to the average total costs.

Tables 4, 5 present the mean scores of the independent to minimally dependent in ADL group, the moderate to maximally dependent in ADL group, mean difference and significance level between the subgroups for SF-36 and SARA. We found a significant difference between the disease severity scores of the two subgroups.

**Table 4:** Mean scores of QoL reported using SF-36 (italics text).

**QoL domains**	**Independent to**	**Moderate to**	**Total**	***p*-value**
	**minimal**	**maximal**		
	**dependent**	**dependent**		
	**mean (SD)**	**mean (SD)**	**mean (SD)**	
Physical functioning	45.0 (32.9)	23.7 (16.2)	26.5 (19.8)	0.042^*^
Role (physical health)	18.3 (14.05)	20.2 (31.8)	17.6 (30.4)	0.087
Role (emotion)	41.7 (41.9)	55.6 (47.1)	53.8 (46.1)	0.680
Energy	36.3 (22.9)	57.0 (22.8)	54.4 (23.5)	0.155
Emotional well-being	61.0 (25.0)	76.0 (21.0)	74.1 (21.7)	0.235
Social functioning	50.0 (22.8)	56.5 (37.6)	55.6 (35.8)	0.654
Pain	41.9 (26.0)	68.5 (31.2)	65.1 (31.6)	0.094
General health	51.3 (24.3)	40.5 (21.0)	41.9 (21.3)	0.374
Health change	37.5 (25.0)	24.6 (19.7)	26.3 (20.5)	0.350

* *p < 0.05 Indicates statistical significance*.

**Table 5:** Mean scores of disease severity due to cerebellar ataxia reported using SARA.

**SARA items**	**Independent**	**Moderate to**	**Total**	***p*-value**
**(score range)**	**to minimal**	**maximal**		
	**dependent**	**dependent**		
	**Mean (SD)**	**Mean (SD)**	**Mean (SD)**	
Gait (0–8)	3.0 (2.2)	6.2 (1.3)	5.8 (1.8)	0.003^*^
Standing (0–6)	1.3 (1.0)	4.2 (1.7)	3.8 (1.9)	0.006^*^
Sitting (0–3)	0.0 (0.0)	1.4 (1.6)	1.2 (1.6)	0.056
Speech Disturbance (0–6)	0.3 (0.5)	2.4 (1.6)	2.1 (1.6)	0.006^*^
Finger chase (0–4)	1.1 (0.8)	1.7 (0.8)	1.6 (0.8)	0.210
Nose-finger test (0–4)	0.4 (0.3)	2.0 (1.1)	1.9 (1.2)	0.013^*^
Alternating movements (0–4)	0.5 (0.7)	2.8 (0.6)	2.5 (1.0)	<0.001^*^
Heel shin (0–4)	0.8 (0.3)	2.5 (1.1)	2.2 (1.2)	0.003^*^
Total score	7.3 (2.2)	23.2 (6.5)	21.2 (8.2)	<0.001^*^

* Significant p < 0.05. *p < 0.05 indicates statistical significance.

Table 6 reports the correlation amongst QoL, disease severity and cost. A significant correlation negative was found between the severity of ataxia (SARA) and the following domains of QoL: physical functioning (*r* = −0.58) and general health (*r* = −0.40). For socioeconomic costs, ataxia severity significantly (*p* < 0.05) and positively correlated with direct costs (Spearman's rho = 0.40), the cost of hiring a caregiver (Spearman's rho = 0.43), and home modification (Spearman's rho = 0.37).

**Table 6:** Correlation of ataxia severity with QoL and cost.

**Items**		**Correlation coefficient**	***p*-value**
**Disease severity (SARA) vs. QoL (SF-36)**			
Physical functioning^#^		−0.579	0.001^*^
Role (physical health)^∧^		0.095	0.612
Role (emotion)^∧^		−0.079	0.672
Energy^#^		0.008	0.964
Emotional well-being^#^		−0.245	0.184
Social functioning^∧^		−0.133	0.475
Pain^∧^		0.181	0.329
General health^#^		−0.407	0.023^*^
Health change^∧^		−0.333	0.067
**Disease severity (SARA) vs. cost**			
Direct cost	In-patient care^∧^	0.329	0.071
	Out-patient care^∧^	0.339	0.062
	Drugs^∧^	0.110	0.555
	Rehabilitation^∧^	0.263	0.153
	Walking aids^∧^	0.384	0.033^*^
	Miscellaneous^∧^	−0.022	0.183
	Total^∧^	0.390	0.030^*^
Indirect cost	Caregiver^∧^	0.432	0.015^*^
	Transportation^∧^	0.279	0.128
	Home modification^∧^	0.371	0.040^*^
	Production loss^∧^	0.205	0.268
	Total^∧^	0.246	0.183
Overall expense^∧^		0.318	0.081

* *Significant p < 0.05*,

# *Pearson's r*,

∧ *Spearman's rho*.

### Sensitivity Analyses

**Figure 1:**
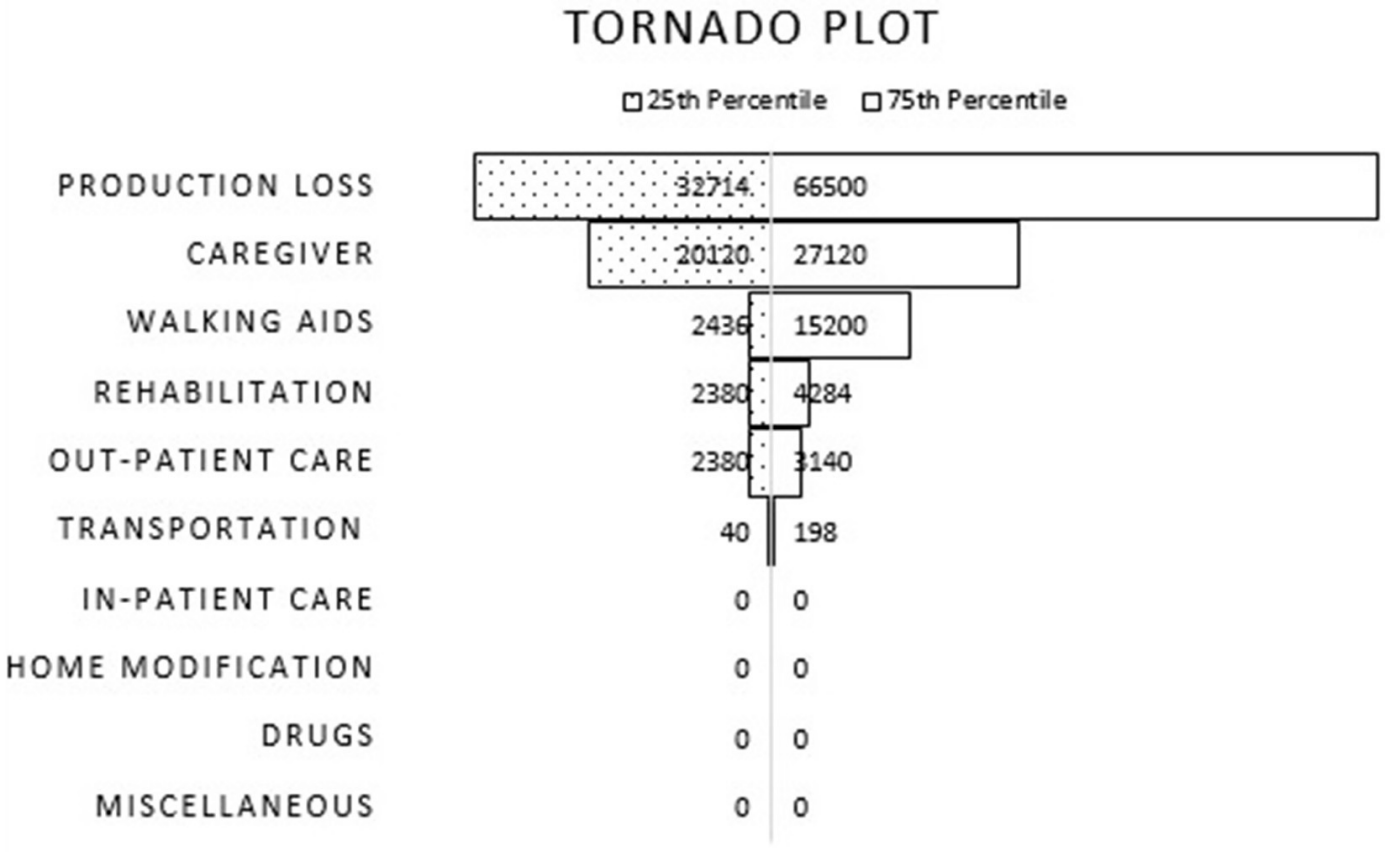
Tornado plot illustrating impact of each cost item toward the total cost at 25th and 75th percentile.

In the first adjusted analysis we removed the data of 2 patients with acquired cerebellar ataxia (stroke and CP). Appendix 1 summarizes the findings of the first sensitivity analysis. The findings of this analysis did not show any important difference. Figure 1 illustrates the findings of the second sensitivity analysis, the Tornado plot. The cost item “productivity loss” had the largest impact on the cost followed by “cost for hiring care-giver” and “walking aids” at the 25th and 75th percentile.

## Discussion

The present study aimed to determine the socioeconomic costs of patients with cerebellar ataxia in Hong Kong for the base year 2019. The average estimated costs for each patient with cerebellar ataxia was HKD 146,832 (HKD 70956 and HKD 158073 for independent to minimally dependent patients and moderate to maximally dependent patients, respectively) for 6 months. Direct costs accounted for 35% of the expenses, and indirect costs accounted for 65%. Patients indicated that access to visiting specialized consultants is limited, and most outpatient visits are made in general outpatient clinics. Besides, the number of visits per week to rehabilitation services, such as physiotherapy, occupational therapy and speech therapy is limited to once in most cases. Additional support in terms of employment following the onset of ataxia can be considered for the target population to address the significant production loss (average of 42%) in patients with cerebellar ataxia given the possibility of the patients losing their ability to work. Furthermore, nearly all the patients had one or more falls in the last 6 months and the majority of the patients with cerebellar ataxia (87%) are frequent fallers.

The symptoms of cerebellar ataxia affect patients physically and psychologically, substantially influencing the economic burden of the patients and the country. To date, only one study on full economic and QoL evaluation is available globally (6). The findings of our study are in-line with the finding of the previous study conducted among 84 patients with spinocerebellar ataxia in Spain (6) in that, they also reported productivity loss due to early retirement and cost of informal care as the leading categories for the disease cost. No socioeconomic cost study among patients with cerebellar ataxia has been conducted in Hong Kong or other regions within Asia. Therefore, previously available cost findings may be used as a reference. Comparing the cost findings of our study with the previous study is limited due to the individual differences in the healthcare system, culture, healthcare policies, the resources of each country and the base year of cost estimation (22). In contrast to the previously published study (6), we used SARA to estimate disease severity. SARA is a reliable, valid and sensitive measuring tool for estimating disease severity amongst patients with cerebellar ataxia (9, 10). Besides, we also used SARA scores to subcategorise our patients based on their independence in ADL, which arguably exhibits better accuracy. A study published in China which has a comparable healthcare system to that of Hong Kong reported the annual cost of Parkinson's disease as HKD 24,995 (currency converted to base-year 2019) (23). The estimated lower cost for patients with Parkinson's disease could be due to the lower contribution of productivity loss as Parkinson disease is common among older adults who may have retired during disease onset and secondly, we estimated cost based on unsubsidized charges while they estimated the actual amount paid by the patient with Parkinson's disease in China. Set aside the individual differences in the cost estimation, in general, the cost of cerebellar ataxia appears to be higher than Parkinson's disease in this region.

Eligible citizens of Hong Kong receive 88% subsidy (18) on all the items listed under direct cost and a one-off subsidy for home modification alone under the indirect medical cost items. This approximates to a mean cost of HKD 45,206 paid by the healthcare system of Hong Kong and the remaining HKD 6,165 is paid by the patient toward direct medical cost over 6 months. Amongst indirect expenses, the cost of hiring a caregiver accounts for the second-highest percentage (17%) of the total costs following production loss. At present, patients with severe cerebellar ataxia hire a minimum of one full-time domestic helper for daily care. The salary of a full-time domestic helper ranges from HKD 4520 to HKD 6,000 per month. Support systems, such as subsidies for paying domestic helpers or employing nurses, funded by the government for wheelchair-bound and bedridden patients with cerebellar ataxia is recommended.

Amongst the nine domains of QoL, six did not correlate with disease severity (SARA). The possible reason for this result can be explained in terms of the limitations of the properties of the measurement tool. Although SARA has been extensively validated for estimating disease severity (24), this measure may not adequately reflect disease severity in certain diseases with extracerebellar features. SARA rates symptoms related to ataxia but does not consider non-ataxia symptoms that are common in ataxia patients, particularly those with spinocerebellar ataxia (9). Hence, if the patient's QoL is influenced by non-ataxic symptoms, then the SARA score may not correlate well with the results of SF-36. By contrast, based on the present findings, it could be argued that disease severity does not impact the perceived QoL in patients with cerebellar ataxia.

Patients who are independent to minimally dependent in ADL (*n* = 4) reported continuing employment with reduced working hours. The mean production loss amongst these patients was HKD 8733. Amongst the moderate to maximally dependent in ADL group (*n* = 27), seven reported inability to work. The mean loss of productivity amongst this group of patients was found to be HKD 69584. Notably, 84% of patients with severe disease retire early due to cerebellar ataxia. Only productivity loss in the past 6 months was considered in this study. The overall mean loss was found to be HKD 60,850. Lifetime production loss may truly reflect between-group difference. Productivity loss increases the economic burden amongst these patients.

The expenditure on housing and home modifications accounted for a mean cost of HKD 2141 per 6 months. Amongst the 31 patients, 17 lived in public housing provided by the Hong Kong government. Notably, 76% of the patients required home modifications, such as installing handrails and ramps. As reported by the patients, these charges were waived by the Hong Kong Housing Authority. Patients with more severe disease spend five times more on home modifications than patients in the less severe group.

HKSCAA, a self-help group for patients with cerebellar ataxia in Hong Kong, provides support and guidance for patients and their caregivers. The association conducts regular meetings and activities for patients, allowing them to socialize with peers within the community. HKSCAA also acts as an intermediary between patients and the government by educating patients on available resources. In this study, we did not include the socializing costs of patients because such costs are not standardized. A negative correlation was anticipated between disease severity and the social functioning item of SF-36. However, an insignificant correlation was found between these items (*p* = 0.431). Such insignificance can be potentially due to the small sample size. Approximately 70% of the participants responded that interference occurs in social activities. This interference indicates that restrictions in physical health obstruct social activities. Therefore, the government and non-governmental organizations are recommended to provide the required support for involving patients in social events that will improve and enhance community participation.

Our study demonstrates the following strengths: (1) To the best of our knowledge, this study is the first of its kind in Asia. (2) The rigorous steps involved in cost estimation using the best practice for estimation (16) by establishing the standard unit price add strength to the findings of this study. (3) The participants were recruited through HKSCAA, reflecting true representation of the community in Hong Kong. (4). Standardized assessment tools that are specific to cerebellar ataxia were utilized to estimate disease severity (SARA) and QoL (SF-36). (5). Lastly, we used the Chinese translated version of SF-36 and the standard questionnaire to obtain a more accurate response from our participants.

Several limitations are observed in this study. Firstly, a small sample size of 31 patients was recruited, which might have compromised the power of the study. The HKSCAA is the only special interest group for patients with cerebellar ataxia in Hong Kong. The HKSCAA has 126 patients registered with a confirmed diagnosis of cerebellar ataxia. Our sample represents 25% of the confirmed cases in Hong Kong and may be considered a representative sample for the patients with cerebellar ataxia in Hong Kong. In addition, given that Hong Kong is a small city with a low prevalence rate of ataxia, 31 participants may be considered acceptable. However, future studies in this region may include data from neighboring countries, such as China, to recruit a larger sample size. Furthermore, all the patients were recruited through HKSCAA, which is the only special interest group for patients with cerebellar ataxia in Hong Kong. Future studies may consider recruiting potential participants through neurology outpatient units in Hong Kong public hospitals. Secondly, the measurement tools used in our study consisted of self-reported questionnaires. Therefore, the patients might not have fully understood the wordings and made less accurate choices. However, we used the standardized Chinese version of the self-rated SF-36 questionnaire to prevent compromising the internal validity of the findings. Thirdly, the assessment of cerebellar ataxia severity was conducted by more than one assessor. However, inter-rater reliability between the assessments was ensured because all the examiners involved in the assessment were trained by the same experienced researcher. Lastly, recall bias is inevitable when completing the cost items because this study was retrospective. A retrospective analysis was planned due to limitations in time and resources. We recommend future studies to conduct prospective analysis for a more accurate daily recording of expenditures. Despite these limitations, the findings of this study may be considered a prelude to future large cost-utility studies amongst patients with cerebellar ataxia.

## Conclusion

The mean cost for patients with cerebellar ataxia for 6 months in Hong Kong is HKD 146,832. The majority of the costs accounted for indirect expenses due to the disease. Additional support in terms of employment, access to specialist consultants and informal home care and community participation are some areas that should be addressed. A future study on a larger population with a prospective design is necessary to confirm the aforementioned claims.

## Data Availability Statement

The datasets generated for this study are available on request to the corresponding author.

## Ethics Statement

The studies involving human participants were reviewed and approved by Human Subjects Ethics Committee of the Hong Kong Polytechnic University (HSECS reference number: HSEARS20190524001). The patients/participants provided their written informed consent to participate in this study.

## Author Contributions

WS was involved in developing the concept, preparation of the study methodology, statistical analysis, and drafting the manuscript. CK, CT, TL, TR, and YH were involved in the data collection, statistical analysis, and drafting of the manuscript. RC was involved in statistical analysis and drafting the manuscript. All authors contributed to the article and approved the submitted version.

## Conflict of Interest

The authors declare that the research was conducted in the absence of any commercial or financial relationships that could be construed as a potential conflict of interest.
