# Supplementary material for: Cost of Cerebellar Ataxia in Hong Kong: A Retrospective Cost-of-Illness Analysis
Source: Front Neurol. 2020 Jul 17;11:711. doi: 10.3389/fneur.2020.00711 (PMC7380245; doi:10.3389/fneur.2020.00711)
Supplement: Supplementary file 1 [file Data_Sheet_1.docx]

Appendix 1. The mean cost (SD) [% of total cost] of patient with cerebellar ataxia (n=29) excluding those with acquired cause (Stroke and Cerebral palsy) in the past 6 months. (in HKD)

| Cost items | Disease severity | | | Average cost  Mean (SD) in HKD |
| --- | --- | --- | --- | --- |
|  | Independent to minimal dependent  Mean (SD) in HKD | Moderate to maximal dependent  Mean (SD) in HKD | Mean difference | Total  Mean (SD) |
| Total cost | 70956.2 (52825.2) | 164184.4  (150296.1) | 93228.2 | 151325.4 (143983.6) |
| Direct cost | 21906.7 (21117.8) [30.9%] | 56168.7  (68359.4) [34.2%] | 34262.0 | 51442.9 (64790.3) [34.0%] |
| In-patient care | 307.5  (615.0)  [0.4%] | 26582.4  (64099.3) [16.2%] | 26274.9 | 22958.3  (60056.8) [15.2%] |
| Out-patient care | 1815.0 (2288.1) [2.6%] | 4061.6 (3232.3) [2.5%] | 2246.6 | 3751.7 (3184.0) [2.5%] |
| Drugs | 6670.7 (13341.4) [9.4%] | 5285.0 (13436.8) [3.2%] | 1385.7 | 5476.1 (13193.3) [3.6%] |
| Rehabilitation | 7140.0 (14280.0) [10.1%] | 12138.0 (25227.3) [7.4%] | 4998.0 | 11448.6 (23883.6) [7.6%] |
| Walking aids | 3973.5 (7491.4) [5.6%] | 9347.2 (7216.3) [5.7%] | 5373.7 | 8606.0 (7362.4) [5.7%] |
| Miscellaneous | 2000.0 (4000.0) [2.8%] | 704.8 (1106.7) [0.4%] | 1295.2 | 883.4 (1723.6) [0.6%] |
| Indirect cost | 49049.5 (54445.5) [69.1%] | 106025.4 (111635.0) [64.6%] | 56975.9 | 98166.7 (106768.1) [64.9%] |
| Caregiver | 6780.0 (13560.0) [9.6%] | 30374.4 (22581.9) [18.5%] | 23594.4 | 27120.0 (22920.6) [17.9%] |
| Transportation | 500.0  (707.1)  [0.7%] | 397.8 (583.3) [0.2%] | 102.2 | 411.9  (588.6) [0.3%] |
| Home modification | 578.0  (719.1)  [0.8%] | 2562.4 (2462.4) [1.6%] | 1984.4 | 2288.7 (2395.4) [1.5%] |
| Production loss | 8733.0 (11408.0) [12.3%] | 72690.8 (98900.9) [44.3%] | 63957.5 | 63869.1 (94349.1) [42.2%] |
